# Prelinguistic intersubjective and socio-communicative skills in infants with neurodevelopmental disabilities aged 0–36 months: A new assessment and parent support tool

**DOI:** 10.3389/fresc.2023.1088853

**Published:** 2023-02-02

**Authors:** Sandra Strazzer, Daniela Sacchi, Roberta Rigamonti, Annalisa Miccoli, Margherita Bonino, Serena Giancola, Chiara Germiniasi, Rosario Montirosso

**Affiliations:** ^1^ Neurophysiatric Department, Scientific Institute IRCCS “Eugenio Medea”, Bosisio Parini, Lecco, Italy; ^2^ 0-3 Center for the at-Risk Infant, Scientific Institute IRCCS “Eugenio Medea”, Bosisio Parini, Lecco, Italy

**Keywords:** neurodevelopmental disabilities, early intervention, intersubjectivity, socio-communicative skills, parental support

## Abstract

**Background:**

Although children with neurodevelopmental disability (NDD) present with several deficits, they partially share developmental impairments in prelinguistic intersubjective and socio-communicative skills, which are not easily assessed by conventional tests during the first years of life.

**Aim:**

The current paper presents a new procedure to assess the prelinguistic intersubjective and socio-communicative skills of NDD children aged 0–36 months. A specific observation form template, called the Observation of Prelinguistic Intersubjective and Socio-Communicative Skills (OPISCoS) form, has been designed to systematically detect infant skills during daily routines (e.g., mealtime, playtime, desk activities). The OPISCoS form helps speech therapists to provide parents support to better perceive and understand early communicative signals from their children, avoiding the risk of excessive or reduced social stimulation.

**Methods:**

The OPISCoS form is composed of three sections, namely, “Pragmatics and Communication,” “Decoding,” and “Expression,” which are useful to delineate the communication abilities of children with NDD and are not tapped by traditional batteries. Vignettes from clinical practice illustrate and provide exemplifications for using the OPISCoS form with NDD infants and their parents.

**Results:**

The OPISCoS form was reported for two children and showed potential in detecting disrupted communicative behaviors and planning specific early interventions. Further, we observed an improvement not only in children's communicative abilities improve but also in their interactions with parents. From a clinical point of view, the OPISCoS form (1) offers an observational perspective of prelinguistic intersubjective and socio-communicative skills in infants with NDD and (2) may be useful to practitioners to enhance parents’ sensitivity to their infants’ communicative behavior.

**Conclusion:**

The OPISCoS form was developed in clinical practice and is based on a very preliminary description of a new observational procedure as integration for the assessment of NDD children. The OPISCoS form appears to be a useful tool for the clinical assessment of prelinguistic intersubjective and socio-communicative skills in NDD infants as well as for promoting the quality of early parenting.

## 1. Introduction

Every year millions of children worldwide receive a diagnosis of neurodevelopmental disability (NDD) (1), with a prevalence rate fluctuating globally between 4.70% and 88.50%, as reported in a systematic review published in 2022 (2). Such a great variation in percentage seems to be ascribable to both estimation procedures and socio-contextual factors (2). Nonetheless, in 2016, NDD was diagnosed in 13.3% of children younger than 5 years in 195 countries (1). The 2021 Annual Report from the Centers for Disease Control and Prevention in the United States estimated that one in six children (16.6%) had a developmental disability or a developmental delay (www.cdc.gov/ncbddd/aboutus/report/).

NDD is a broad clinical label including diverse clinical conditions ranging from impairments in vision and hearing to intellectual disabilities, from infant cerebral palsy to genetic syndromes, and from serious brain injuries to conduct disorders (3). Children with NDD may display an array of impairments in several developmental domains, including cognitive, behavioral, motor, and emotional functioning, and frequently show an altered capacity to cope with daily stress (2, 3). Furthermore, they have been found to exhibit lower interactive and attentional skills and use less vocal and affective signals and also display behavioral instability in day-to-day exchanges with parents (4, 5). Such altered behavioral and socio-emotional patterns, along with the fact that parents are required to deal with an unknown world made of new rules, decisions, and barriers, may negatively impact parental psychological wellbeing and the quality of the early parent–child relationship (6–8). Previous research highlighted that early parenting support interventions are recommended to promote adequate parent–child interaction and, as a consequence, infants’ developmental outcomes (e.g., receptive and expressive language, or even motor developmental outcome), even in the case of NDD (9–15). Early interventions are defined as “multidisciplinary services provided to children from birth to 5 years of age to promote child health and well-being, enhance emerging competencies, minimise developmental delays, remediate existing or emerging disabilities, prevent functional deterioration and promote adaptive parenting and overall family function” [(16), page XVII]. Assisting families of children with NDD through parenting interventions allows caregivers to better understand infants’ signals, enabling an appropriate response and providing suitable stimulations (5, 7). The research documented how early interventions geared to actively involve parents in informative and psycho-educational programs attenuate long-term negative effects of child's disabilities (5, 13, 17, 18). Early interventions seem to be more effective when carried out in approximately the first thousand days of life owing to rapid brain growth and remarkable neuroplasticity, with cascade effects on child development (15, 19, 20).

Among the various experiences that stimulate and model the child's brain structures and functions involved in cognitive and communication development, a relevant role is played by contingent interactions between the child and caregivers (21). Importantly, a child's early language experiences predict school-age language and developmental outcomes years later (22). Participation in early social interactions is crucial to stimulate language, and scholars suggest that primary and secondary intersubjectivity are precursors to the emergence of verbal communication (23). Primary intersubjectivity refers to mutual emotional and attentional coordination between infant and mother during face-to-face exchanges, a dyadic experience that begins in the child's first days of life. Secondary intersubjectivity refers to triadic coordination, arising around age 9 months; it allows the infant’s and mother's shared attention to specific characteristics of the environment they jointly attend. It implies a cooperative exchange of referential gestures between the infant and mother and is critical for the infant's ability to utter their first words at around 12 months (24, 25). In sum, intersubjectivity could play a key role in the development of intentional communication in infants (26). Indeed, during the bidirectional and contingent communication between infants and mothers, caregivers use verbal and nonverbal inputs (e.g., pointing) and pragmatic strategies (e.g., labeling, imitation of the child's utterance, expansion, and interpretation of their vocalization) to sustain the child's shift from prelinguistic to linguistic communication and enhance language development (27, 28). Thus, when the adult's child-directed speech is contingent, timely, and suitable to the child's communicative interactions, both temporally and conceptually, it enhances the relevance of the information and the probability of linking the specific event to the words increasing the association between language content, form, and use, thus limiting the infant's cognitive load (29–32).

There is limited knowledge on the role of prelinguistic intersubjective skills in cognitive and communication ability of children at risk of language impairments, such as NDD. According to a review (33), caregivers of children with clinical conditions tend to adopt more intrusive and directive speech and, without clinical support, they do not spontaneously improve the quality of this speech, possibly limiting children's language improvement. On the other hand, “mother fluency and connectedness”—a measure of interaction quality that captures the flow and cohesion between mother and child, was associated with better language skills at the age of 24 months, and excessive stimulation of children has been associated with nonoptimal language development (11). Specifically, parental communicative behaviors, such as intrusive directives, resulted in a mismatch between unsuccessful stimulation and the child's competency level, which increased avoidance behaviors in children (11).

On the other hand, contingent interactions between the child and parents can be difficult when the infant has some neurodisability due to unclear or unintelligible communicative behavior. Parents could not be able to interpret infants’ cues and communications because of the partial or reduced signals like facial expressions, pointing, reaching, showing objects, and so on, which in turn could make it difficult for parents to understand their infant's intention (4, 5, 34). Primary intersubjectivity allows the infant to organize contingent exchanges with the parent, specifically in terms of protoconversion, which includes a variety of communication forms (23). Consequently, altered prelinguistic intersubjective skills, such as deficits in vocalizations, eye contact, smiling, and sharing attention, may interfere with the quality of daily dyadic exchanges, which in turn may negatively affect parenting with a long-term negative impact on cognitive and language development (35).

The above-mentioned evidence shows that prelinguistic intersubjective skills (both primary and secondary) are an important target for early intervention programs in the context of NDD. Unfortunately, to the best of our knowledge, there are no studies on observational procedures aimed at examining prelinguistic intersubjective and socio-communicative skills in children with NDD, which could possibly be used to promote parents’ competencies during daily exchanges with their infants. Several measures to assess communication in young children with atypical development or at-risk children are currently available, such as the MacArthur–Bates Communicative Development Inventory [MCDI (36)], the Ages and Stages Questionnaire (ASQ) (37), and the Early Communication Indicator [ECI; (38)]. The MCDI and the ASQ are not observational instruments, and the ECI is a play-based assessment administered by a practitioner observing the child playing with a familiar adult partner and using a standard toy set. These tools rely on parent reports or use a specific setting rather than direct assessment in an early rehabilitation setting. For these reasons, they are inappropriate for our clinical purposes and rehabilitation programs for children with NDD.

The main goal of the current paper is to present a new procedure to assess prelinguistic intersubjective and socio-communicative skills in NDD children aged 0–36 months in an early rehabilitation setting. A specific observation form template, called the Observation of Prelinguistic Intersubjective and Socio-Communicative Skills (OPISCoS) form, was designed to systematically detect infant skills during daily routines (e.g., mealtime, playtime, desk activities). The OPISCoS form also helps speech therapists to provide parents support to better perceive and understand early communicative signals of their children, avoiding the risk of excessive or reduced social stimulation. Two case studies will be presented to illustrate the use of the OPISCoS form.

## 2. Materials and methods

### 2.1. OPISCoS form

The OPISCoS form and the associated treatment described in the present paper were developed by a multidisciplinary team working in a child's rehabilitation center in northern Italy. Infants are hospitalized with their parents for several days and take part in a diagnostic and/or rehabilitation daily program (i.e., speech therapy, physical therapy, etc.). During hospitalization, a multidisciplinary team follows the infants and their parents using a family-oriented approach. Depending on the child's performance level and chronological age, speech therapists commonly use a number of tests to assess children's communicative abilities. However, sensory problems, limited attentional abilities, and poor expressive skills of children with NDD (e.g., some children do not utter intelligible sounds and have not learned the “yes/no” code) often make it impossible to use conventional assessment tests. This frequently causes delays in testing, with a negative cascade of effects, for example, lack of access to adequate interventions to improve their disrupted socio-communicative abilities. To get a reliable picture of prelinguistic intersubjective and socio-communicative skills, speech therapists in our team conduct systematic observations of the child's behavior during daily routines using the OPISCoS form. The global picture of children's skills rests on three observations in different contexts resembling everyday activities (e.g., mealtime, playtime). During such observations, speech therapists do not focus solely on child assessment but also provide parental support.

The OPISCoS form is not intended to be used as a standardized index of the functioning or as an indication of the age of competence in the area of communication as established by traditional batteries/tests. It should rather be considered as means to get indications of abilities yet to be developed in children with NDD to work on through *ad hoc* rehabilitation. It provides clinicians with a structured checklist to evaluate specific communication behaviors, act on skills yet to be acquired or to become established, and evaluate improvements in each behavior postintervention.

A description of the OPISCoS form is provided in the paragraph below.

The OPISCoS form is an observational procedure useful to examine prelinguistic intersubjective and socio-communicative skills in children with NDD from birth to age 36 months. It includes an evaluation of all communicative abilities expected to develop in a typically developing child in the target age range, such as the distinction between deictic gestures and representative gestures. It can also be used to complement the assessment of NDD children, who are only partially assessable through conventional tests. The OPISCoS form consists of three sections:

1) *Pragmatics and Communication*. This section is aimed at evaluating (A) Interaction and Communicative Acts, such as eye contact, facial expressions, vocalizations, smiles, etc.; (B) Intersubjectivity, as expressed in communicative intentionality (i.e., declarative pointing), Attention (dyadic and triadic attention), Turn-taking and Social Conversation Skills (responsiveness and assertiveness); (C) Communication Style (if the child appears involved or passive in interaction); and the (D) Modifiability to the speech therapist's proposal.
2) *Decoding*. This second section deals with decoding abilities associated with infants’ attention to sounds and reaction to their name and their caregiver's name; abilities defined here are (A) First signals of comprehension; (B) Affirmation/Negation; (C) Comprehension of motor or verbal routines, up to an understanding of word meaning; and (D) Recognizing.
3) *Vocal production*. The child's expressive communication skills are evaluated in the following order, according to age: (A) Vocalizations; (B) Babbling; (C) Word production; (D) Sound effects and animal sounds; (E) Phonetic processes; (F) Phonological processes; and (G) Modifiability to the therapist's proposal.

The OPISCoS form is shown in Table 1.

**Table 1:** Observation of the Prelinguistic Intersubjective and Socio-Communicative Skills (OPISCoS) form.

1. Pragmatic and Communication		
Subdomain	Item	Code
Interaction and communicative acts	Eye contact	Present
		Partially present
		Absent
	Mimic modulation	Present
		Partially present
		Absent
	Smile	Present
		Partially present
		Absent
	Cry	Present
		Partially present
		Absent
	Attention getting	Present
		Partially present
		Absent
	Eye contact in joint attention	Present
		Partially present
		Absent
	Voice communication	Present
		Partially present
		Absent
	Communicative gesture	Present
		Partially present
		Absent
	Vocalization + communicative gesture	Present
		Partially present
		Absent
Communicative intentionality	*Deictic gesture*
	Imperative pointing	Present
		Partially present
		Absent
	Declarative pointing: Show	Present
		Partially present
		Absent
	Declarative pointing: Give	Present
		Partially present
		Absent
	*Representative gesture*
	Social regulators (e.g., yes/no; hello, good)	Present
		Partially present
		Absent
	Iconic (e.g., eating)	Present
		Partially present
		Absent
	Personal (e.g., gestures learned by themselves, with social meaning)	Present
		Partially present
		Absent
Attention	Dyadic	Present
		Partially present
		Absent
	Triadic with joint attention	Present
		Partially present
		Absent
	Sustained (e.g., activities, environment)	Present
		Partially present
		Absent
Turn-taking		Present
		Partially present
		Absent
Social conversation skills	Responsiveness	Present
		Partially present
		Absent
	Assertiveness	Present
		Partially present
		Absent
Communication style	Involved/Active	Present
		Partially present
		Absent
	Involved only if motivated	Present
		Partially present
		Absent
	Passive/Need to solicit (response to therapist's proposal)	Present
		Partially present
		Absent
	Dysregulated	Present
		Partially present
		Absent
Modifiability to the therapist's proposal (Pragmatics and Communication)		
Adaptability to new strategies proposed by the therapist	Present
Partially present
Absent
Imitation	Present
Partially present
Absent
2. Decoding		
Subdomain	Item	Code
First signals of comprehension	Sound source orientation	Present
		Partially present
		Absent
	Response to his/her name	Present
		Partially present
		Absent
	Reaction to the caregiver's name	Present
		Partially present
		Absent
	Reaction to “No”	Present
		Partially present
		Absent
Affirmation/negation	Contextual Yes/No	Present
		Partially present
		Absent
Comprehension of motor routines	Greeting, clapping, blowing a kiss, opening mouth, caressing	Present
		Partially present
		Absent
Comprehension of verbal routines	Still/enough, Give me…, Look, Where is…? Here we go, Let's go out Drinking/eating Animal sounds	Present
		Partially present
		Absent
Comprehension of the main parts of the body	Mouth, eyes, ears, nose, hair, feet, hands, belly	Present
		Partially present
		Absent
Recognizing	Lexical (by name)	Present
		Partially present
		Absent
	Syntactic (by use)	Present
		Partially present
		Absent
	Simple orders (e.g., feed the doll)	Present
		Partially present
		Absent
3. Vocal production		
Subdomain	Item	Code
Expression	Vocalizations	Spontaneous
		Repetition
		Absent
	Babbling Canonical	Spontaneous
		Repetition
		Absent
	Babbling Varied	Spontaneous
		Repetition
		Absent
	Sound effects and animal sounds	Spontaneous
		Repetition
		Absent
	Words Disyllabic	Spontaneous
		Repetition
		Absent
	Words Trisyllabic	Spontaneous
		Repetition
		Absent
	Words Multisyllabic	Spontaneous
		Repetition
		Absent
	Phonetic inventory, Age-appropriate	Yes
		No
	Phonological processes, age-appropriate	Yes
		No
	Intelligibility	Yes
		No
	Prosody	Yes
		No
Modifiability to the therapist's proposal (Vocal production)		
Adaptability to new strategies proposed by the therapist	Present
Partially present
Absent
Imitation	Present
Partially present
Absent

#### 2.1.1. How to use the OPISCoS form

Each observation lasts at least 30 min to collect enough data about the three areas of communication skills (i.e., Pragmatic and Communication, Decoding, and Vocal production). The observation is conducted purposefully during daily activities such as mealtime, free play, and desk activities and makes children and parents more comfortable.

For each item of the “Pragmatic and Communication” and “Decoding” sections of the OPISCoS form, the observer uses the following code for each behavior: “Present,” “Partially present,” or “Absent.”

- Present: Definite, strong, or frequent behavior (the behavior is observed for at least 75% of the observation time);
- Partially present: Brief, minor, or emerging behavior (the behavior is not so frequently observed: about 50% of the observation time);
- Absent: No behavior is observed during the observation.

By contrast, for the “Expression” section, two different coding systems are used. In the first half of the section:

- Spontaneous: The target behavior is part of the spontaneous pattern of the child;
- Repetition: The target behavior is shown by imitation;
- Absent: Behavior not shown.

In the second half of the section:

- Yes: Behavior is present;
- No: Behavior is absent.

#### 2.1.2. Clinical criteria underlying the OPISCoS form

The OPISCoS form is intended to be used in clinical practice with children (both in- and outpatients) presenting with neurological conditions, such as acquired or congenital brain injury or other NDDs. The OPISCoS form is suitable for children aged 0–36 months for whom traditional language batteries/tools cannot be used, while it is not recommended for children with low levels of consciousness or with extremely severe (sensory and motor) disabilities.

#### 2.1.3. OPISCoS as a way to support parents with infants with NDD

As mentioned before, assisting caregivers of children with NDD through early parenting interventions allows them to better understand their infant's signals, enabling an appropriate response and a suitable degree of stimulation. The OPISCoS form can be an effective way to improve parental awareness of infants’ signals as they “learn” to help them better read and interpret infant's cues and develop more appropriate communication strategies. To set up the OPISCoS form discussion meetings, speech therapists share with caregivers their rehabilitation goals, as follows: “Now I would like to share with you the OPISCoS form we completed during our observation of your child. You might be interested about some aspects of your infant's communicative behaviour or you may be uncertain the best way to interact with him/her. Thus, feel free to comment on, or ask about any item we go through.” The speech therapists guide the discussion in a nondirective way, supporting the parents’ view and—when appropriate—suggesting new insights. General strategies are adapted specifically to each child. Speech therapists demonstrate these strategies to parents to promote the modeling process so that these behaviors become established in everyday life after hospital discharge. Follow-up assessments are planned over time to update rehabilitation goals.

### 2.2. Procedure

To better illustrate the use of the OPISCoS form, we present two clinical cases. The OPISCoS form was used to evaluate the communication profile and implement the related rehabilitation intervention. For these children, a multidisciplinary daily rehabilitation program requiring hospitalization was planned. In addition to communication, other motor and educational aspects were worked on during hospitalization.

For each case study, clinical and demographic information was collected; the various items of the OPISCoS form were considered at three time points: baseline assessment (prerehabilitation intervention), immediate postrehabilitation assessment, and follow-up assessment (at least 3 months after the intervention).

### 2.3. Case studies

#### 2.3.1. Case study 1

N. was a 21-month-old child with right unilateral infantile cerebral palsy caused by grade 3 intraventricular hemorrhage. The child walks independently but has difficulties with balance, coordination, and speed. According to the Gross Motor Function Classification System (GMFCS) (39), he scored level I. He had greater difficulty with his upper limbs and object manipulation. He also needed to be helped in some activities that needed adaptation to optimize his abilities. He scored level III on the Mini-Manual Ability Classification System (Mini-MACS) (40). His family's socioeconomic status (SES) is “medium business, minor” (41). During his first clinical examination, N. showed communicative intentionality through eye contact and was able to show pleasure by smiling and disappointment through facial expressions. N. used gestures to indicate and call the interlocutor. His attention span was reduced, and dyadic and triadic attention was occasional. N. comprehended motor and verbal routines. He also understood lexical/syntactic decoding of common objects and simple and contextual verbal orders. Yes/No was absent. N. presented lallation and a single word “mom,” which he used as a “passe-partout word.”

His mimic (gestural) abilities appeared to be the most easily modifiable, so the main goal of the intensive treatment was to reinforce these skills. The intervention took place in different settings, such as the play area or the lunch area in the child's room, with the main aim of giving parents a model of interaction during everyday exchanges. During hospitalization, an intensive program was planned (five sessions lasting 45 min each). The speech therapist used the following strategies with N. and also taught them to his mother:

- Stimulation of interaction: voice prompt and tactile contact;
- Joint attention stimulation: positioning of objects/games on the trajectory of glances;
- Prompting of declarative deictic gestures (e.g., show, give): use of gestures as models for the child. For example, the speech therapist asked the child to play by showing him his hand with the palm up and saying “Give it to me”;
- Introducing simple gestures with meaning: the speech therapist and the parent used and asked the child to use a few gestures to support the communicative intent in different daily routines;
- Verbal stimulation: offering a combined vocal/gestural model contextual to the proposed activity.

#### 2.3.2. Case study 2

S. was 35 months old when he first came to our clinical unit with severe traumatic brain injury (TBI) [Glasgow Coma Scale (42) = 5] caused by a severe fall at age 32 months. Her neuroradiological picture was characterized by diffuse axonal damage, with areas of frontal and bilateral cortico-subcortical signal impairment. Although S. had a severe cognitive and communicative impairment, her consciousness was not impaired, as shown by her Coma Near Coma Scale score (43). S. had good left arm motility, but she showed right arm palsy. During her first hospitalization, in the postacute phase, the child learned to walk and uses her left upper limb well but does not use her right limb. Owing to her severe cognitive-linguistic impairment, the girl could not receive a standardized cognitive assessment. An evaluation was performed using the Functional Independence Measure for children (WeeFIM) (44). The WeeFIM scores ranged from 18 to 30; while prevalent improvement in motor skills was observed, she showed minimal-to-absent recovery of cognitive and autonomy skills. A score of 30 means that the child needs help performing different daily tasks and is regarded as “dependent.” Her family SES was “unskilled workers.”

At the end of weaning (within the first month), she established eye contact only with the caregiver and also exhibited vocalizations of pleasure and distress. An intensive intervention program directed to communication (eight sessions lasting 45 min each) was planned with the following aims: to increase eye contact, stimulate joint attention and mimic modulation, and promote pointing through gestures, vocalization, and gestures with meaning (i.e., with motor routines). The speech therapist worked on the following strategies and also taught them to S.’s mother:

a) sustaining triadic joint attention during playtime, using games;
b) giving vocal and gestural modeling, using books with sound effects and animal sounds and first simple words.

## 3. Results

### 3.1. Case study 1

#### 3.1.1. Postintervention assessment

At the end of the intensive intervention program, N. had acquired nodding to the Yes signal, which was used in an appropriate and contextual way. N. also learned to use a repertoire of gestures in response to the stimuli proposed by the interlocutor, which he also did spontaneously. At the expressive vocal level, he had extended his range of sounds and syllables, produced in the form of varied lallation and articulatory play or to support the gestural request of a game. Occasionally, a few sound effects and animal sounds appeared, but no new words were recorded. N. showed no interest in oral–buccal–facial movements and verbal language. Parents were advised to continue stimulating the child at home.

#### 3.1.2. Follow-up assessment

At the 8-month follow-up, N. showed increased deictic gestures to show, request, and draw the interlocutor's attention. The verbal denial code appeared while he kept nodding to signal agreement. Sound effects and animal sounds increased, and a few formulaic expressions and 12 words with consonant–vowel–consonant–vowel structure appeared. N. could not be evaluated by testing as he showed no interest in figurative material. The results of the first intervention and follow-up assessment are shown in Table 2.

**Table 2:** Case #1 OPISCoS form: effects of the intervention at preintervention (T0), immediate postintervention (T1), and follow-up after 8 months (T2).

1. Pragmatics and Communication					
Subdomain	Item	Code	T0	T1	T2
Interaction and communicative acts	Eye contact	Present	X	X	X
		Partially present			
		Absent			
	Mimic modulation	Present	X	X	X
		Partially present			
		Absent			
	Smile	Present	X	X	X
		Partially present			
		Absent			
	Cry	Present	X	X	X
		Partially present			
		Absent			
	Attention getting	Present			X
		Partially present	X	X	
		Absent			
	Eye contact in joint attention	Present			X
		Partially present	X	X	
		Absent			
	Voice communication	Present			X
		Partially present	X	X	
		Absent			
	Communicative gesture	Present	X	X	X
		Partially present			
		Absent			
	Vocalization + communicative gesture	Present			X
		Partially present			
		Absent	X	X	
Communicative intentionality	*Deictic gesture*				
	Imperative pointing	Present	X	X	X
		Partially present			
		Absent			
	Declarative pointing: Show	Present			X
		Partially present			
		Absent	X	X	
	Declarative pointing: Give	Present			X
		Partially present			
		Absent	X	X	
	*Representative gesture*				
	Social regulators (e.g., yes/no; hello, good)	Present			X
		Partially present		X	
		Absent	X		
	Iconic (e.g., eating)	Present			X
		Partially present		X	
		Absent	X		
	Personal (e.g., gestures learned by themselves, with social meaning)	Present			
		Partially present			
		Absent	X	X	X
Attention	Dyadic	Present			X
		Partially present	X	X	
		Absent			
	Triadic with joint attention	Present			X
		Partially present	X	X	
		Absent			
	Sustained (e.g., activities, environment)	Present			
		Partially present			X
		Absent	X	X	
Turn-taking		Present			
		Partially present			X
		Absent	X	X	
Social conversation skills	Responsiveness	Present			X
		Partially present			
		Absent	X	X	
	Assertiveness	Present			
		Partially present			X
		Absent	X	X	
Communicative style	Involved/Active	Present			
		Partially present			X
		Absent	X	X	
	Involved only if motivated	Present			
		Partially present			X
		Absent	X	X	
	Passive/Need to solicit (response to therapist's proposal)	Present	X	X	
		Partially present			
		Absent			X
	Dysregulated	Present			
		Partially Present			
		Absent	X	X	X
Modifiability to the therapist's proposal (Pragmatics and Communication)					
Adaptability to new strategies proposed by the therapist	Present			X
Partially present		X for the mimic component	
Absent			
Imitation	Present			
Partially present			
Absent			
2. Decoding					
Subdomain	Item	Code	T0	T1	T2
First signals of comprehension	Sound source orientation	Present	X	X	X
		Partially present			
		Absent			
	Response to their name	Present	X	X	X
		Partially present			
		Absent			
	Reaction to the caregiver's name	Present	X	X	X
		Partially present			
		Absent			
	Reaction to “No”	Present	X	X	X
		Partially present			
		Absent			
Affirmation/Negation	Contextual Yes/No	Present			X
		Partially present			
		Absent	X	X	
Comprehension of motor routines	Greeting, clapping, blowing a kiss, opening mouth, caressing	Present	X	X	X
		Partially present			
		Absent			
Comprehension of verbal routines	Still/enough, Give me…, Look, Where is…? Here we go, Let's go out Drinking/eating Animal sounds	Present	X	X	X
		Partially present			
		Absent			
Comprehension of the main parts of the body	Mouth, eyes, ears, nose, hair, feet, hands, belly	Present			X
		Partially present	X	X	
		Absent			
Recognizing	Lexical (by name)	Present	X	X	X
		Partially present			
		Absent			
	Syntactic (by use)	Present	X	X	X
		Partially present			
		Absent			
	Simple orders (e.g., feed the doll)	Present	X	X	X
		Partially present			
		Absent			
3. Vocal production					
Subdomain	Item	Code	T0	T1	T2
Expression	Vocalizations	Spontaneous	X	X	
		Repetition			X
		Absent			
	Babbling canonical	Spontaneous	X	X	
		Repetition			X
		Absent			
	Babbling varied	Spontaneous			
		Repetition		X	X
		Absent	X		
	Sound effects and animal sounds	Spontaneous			
		Repetition		X	X
		Absent	X		
	Words disyllabic	Spontaneous	X “mom” as passe-partout word	X “mom” as a passe-partout word	X *N* = 15 words
		Repetition			
		Absent			
	Words trisyllabic	Spontaneous			
		Repetition			
		Absent	X	X	X
	Words multisyllabic	Spontaneous			
		Repetition			
		Absent	X	X	X
	Phonetic inventory, age-appropriate	Yes			
		No	X	X	X
	Phonological processes, age-appropriate	Yes			
		No	X	X	X
	Intelligibility	Yes			X
		No	X	X	
	Prosody	Yes	X	X	X
		No			
Modifiability to the therapist's proposal (Vocal production)					
Adaptability to new strategies proposed by the therapist	Present			X
Partially present			
Absent	X	X	
Imitation	Present			X
Partially present			
Absent	X	X	

### 3.2. Case study 2

#### 3.2.1. Postintervention assessment

After the short rehabilitation intervention, with the mother's training, crying appeared as a means of communication to express “No.” S. began to share her gaze with the therapist. She was not yet able to adapt to new stimuli.

#### 3.2.2. Follow-up assessment

At the 3-month follow-up (T2), S. communicated nutritional needs through complaints, irritability, and motor agitation. Lateral head movement was a signal of satiety and demonstrated self-regulation, too. She showed selectivity for food tastes and consistency in spitting out food but not by mimic modulation. Eye contact had improved; she showed a shared gaze and looked at the caregiver while waiting for an expected stimulus in familiar games. She began to respond to her name and the caregiver's name even when strangers were in the room. Comprehension skills were difficult to assess because of inconsistent responses. While there was an orientation to the sound source, there were only occasional reactions to the interlocutor's voice. S. produced occasional vocalizations. The results of the first intervention and follow-up assessment are shown in Table 3.

**Table 3:** Case #2 OPISCoS form: effects of the intervention at preintervention (T0), immediate postintervention assessment (T1), and follow-up after 3 months (T2).

1. Pragmatics and Communication					
Subdomain	Item	Code	T0	T1	T2
Interaction and communicative acts	Eye contact	Present			
		Partially present			X
		Absent	X	X	
	Mimic modulation	Present			
		Partially present			
		Absent	X	X	X
	Smile	Present			
		Partially present			X
		Absent	X	X	
	Cry	Present			X
		Partially present		X	
		Absent	X		
	Attention getting	Present			
		Partially present			
		Absent	X	X	X
	Eye contact in joint attention	Present			
		Partially present			X
		Absent	X	X	
	Voice communication	Present			
		Partially present			X
		Absent	X	X	
	Communicative gesture	Present			
		Partially present			
		Absent	X	X	X
	Vocalization + communicative gesture	Present			
		Partially present			
		Absent	X	X	X
Communicative intentionality	*Deictic gesture*				
	Imperative pointing	Present			
		Partially present			
		Absent	X	X	X
	Declarative pointing: show	Present			
		Partially present			
		Absent	X	X	X
	Declarative pointing: give	Present			
		Partially present			
		Absent	X	X	X
	*Representative gesture*				
	Social regulators (e.g., yes/no; hello, good)	Present			
		Partially present			
		Absent	X	X	X
	Iconic (e.g., eating)	Present			
		Partially present			
		Absent	X	X	X
	Personal (e.g., gestures learned by themselves, with social meaning)	Present			
		Partially present			
		Absent	X	X	X
Attention	Dyadic	Present			
		Partially present	X caregiver	X	X
		Absent			
	Triadic with joint attention	Present			
		Partially present			
		Absent	X	X	X
	Sustained (e.g., activities, environment)	Present			
		Partially present			
		Absent	X	X	X
Turn-taking	** **	Present			
		Partially present			
		Absent	X	X	X
Social conversation skills	Responsiveness	Present			
		Partially present			
		Absent	X	X	X
	Assertiveness	Present			
		Partially present			
		Absent	X	X	X
Communicative style	Involved / Active	Present			
		Partially present			
		Absent	X	X	X
	Involved only if motivated	Present			
		Partially present			
		Absent	X	X	X
	Passive/Need to solicit (response to therapist's proposal)	Present			
		Partially present			X
		Absent	X	X	
	Dysregulated	Present			
		Partially present			
		Absent	X	X	X
Modifiability to the therapist's proposal (Pragmatic and Communication)					
Adaptability to new strategies proposed by the therapist	Present			X
Partially present		X	
Absent	X		
Imitation	Present			
Partially present			
Absent			
2. Decoding					
Subdomain	Item	Code	T0	T1	T2
First signals of comprehension	Sound source orientation	Present			X
		Partially present		X	
		Absent	X		
	Response to their name	Present			
		Partially present			X
		Absent	X	X	
	Reaction to the caregiver's name	Present			
		Partially present			X
		Absent	X	X	
	Reaction to “No”	Present			
		Partially present			
		Absent	X	X	X
Affirmation/Negation	Contextual Yes/No	Present			
		Partially present			
		Absent	X	X	X
Comprehension of motor routines	Greeting, clapping, blowing a kiss, opening mouth, caressing	Present			
		Partially present			
		Absent	X	X	X
Comprehension of verbal routines	Still/enough, Give me…, Look, Where is…? Here we go, Let's go out Drinking/eating Animal sounds	Present			
		Partially present			
		Absent	X	X	X
Comprehension of the main parts of the body	Mouth, eyes, ears, nose, hair, feet, hands, belly	Present			
		Partially present			
		Absent	X	X	X
Recognizing	Lexical (by name)	Present			
		Partially present			
		Absent	X	X	X
	Syntactic (by use)	Present			
		Partially present			
		Absent	X	X	X
	Simple orders (e.g., feed the doll)	Present			
		Partially present			
		Absent	X	X	X
3. Vocal production					
Subdomain	Item	Code	T0	T1	T2
Expression	Vocalizations	Spontaneous			X
		Repetition			
		Absent	X	X	
	Babbling canonical	Spontaneous			
		Repetition			
		Absent	X	X	X
	Babbling varied	Spontaneous			
		Repetition			
		Absent	X	X	X
	Sound effects and animal sounds	Spontaneous			
		Repetition			
		Absent	X	X	X
	Words disyllabic	Spontaneous			
		Repetition			
		Absent	X	X	X
	Words trisyllabic	Spontaneous			
		Repetition			
		Absent	X	X	X
	Words multisyllabic	Spontaneous			
		Repetition			
		Absent	X	X	X
	Phonetic inventory, age-appropriate	Yes			
		No	X	X	X
	Phonological processes, age-appropriate	Yes			
		No	X	X	X
	Intelligibility	Yes			
		No	X	X	X
	Prosody	Yes			
		No	X	X	X
Modifiability to the therapist's proposal (Vocal production)					
Adaptability to new strategies proposed by the therapist	Present			
Partially present			
Absent	X	X	X
Imitation	Present			
Partially present			
Absent	X	X	X

## 4. Conclusion

The OPISCoS form can help practitioners to get a picture of prelinguistic intersubjective and socio-communicative skills in children with NDD who cannot be tested by conventional measures. At the same time, the OPISCoS form can provide a basis for practitioners to identify which communication behaviors of children should be reinforced or stimulated by *ad hoc* interventions. The OPISCoS form also involves parents in the rehabilitation process through teaching and modeling techniques. Therefore, its use with infants with NDD and their parents in clinical neurorehabilitation settings holds the potential to highlight crucial aspects of infants’ communicative behavior and caregiver–infant interaction.

The two case studies described above show the value of the OPISCoS form in detecting disrupted communication in young children with NDD and enabling specific early interventions to improve the children's communication abilities and, at the same time, to ameliorate parent–child interaction. Its usability makes the OPISCoS form suitable for children with significantly delayed cognitive and communication abilities who cannot be evaluated through conventional tests, thus allowing early detection and remediation of linguistic and interactive deficits. Further, the OPISCoS form enables a comparison between infant's pre- and postintervention acquisitions, highlighting the positive outcomes of early interventions that are not so easily identifiable at first glance. Indeed, modifications often regard minimal behaviors or fully acquired behaviors in young children with NDD.

A key role in the treatment is played by parents. When they receive support, they develop greater awareness of their children's expressive signals, communication timing, and behavioral responses. They become more sensitive to their child's cues, creating interactive patterns with optimal stimulation levels, which, in turn, allows for promoting intersubjective abilities in their child (35). In the clinical cases reported here, at the end of the planned intervention, the two infants exhibited numerous stable acquisitions in the areas of pragmatics and communication, decoding, and expression, which were addressed both by speech therapists during sessions and by parents during everyday activities. Considering that the formal part of the early intervention was limited to only 45 min of the child's awake time during the day, continuous stimulation by the parents all day long appears to have been instrumental to intervention gains.

### Implications for practice

The preliminary observation provided reasons why practitioners working with children with NDD should consider the potential use of the OPISCoS form. For example, the OPISCoS form uniquely supports direct measurement of prelinguistic intersubjective and socio-communicative skills (gestures, vocalization, dyadic attention, etc.) even when they are minimal. Even though further research is needed to support its psychometric properties, the OPISCoS form provides a complete measure of skills that can be captured in the childcare setting by a practitioner who observes a child with NDD. The OPISCoS form is uniquely suited for use in early rehabilitation programs and seems sensitive to the effects of interventions promoting socio-communicative abilities delivered by speech therapists.

Use of tools such as the OPISCoS form is anchored in a wider approach to child development based on the need to deliver interventions that focus on the promotion of parental competencies and resources, above all in the case of children with NDD (45). These interventions are guided by the following assumptions: promotion of responsive parenting plays a key role in the development of and care provided to children; parental support is more effective if performed at a child's earlier age; and interventions to support parents are more useful and effective with children with adverse environmental conditions, such as a clinical diagnosis or at a social disadvantage (46). The more support provided to parents when their child is very young, the better their abilities in the long term, too (46).

### Implications for future research

Research into prelinguistic intersubjective and socio-communicative skills remains a need and a challenge in children with NDD. The OPISCoS form has the potential to fill a gap, but it needs to be tested in randomized studies on specific clinical subgroups (e.g., based on diagnosis or age) to objectively assess its benefits in clinical practice as compared to traditional personalized rehabilitation programs. Also, its psychometric properties, such as interrater reliability and convergent validity, should be investigated. Future work could benefit from efforts to overcome this and the other limitations discussed, including improvements in validation criteria. Clinical studies offer strong approaches to future OPISCoS validity research. For example, the gains in abilities that are yet to be acquired or not fully established at the first OPISCoS evaluation could be compared with the benefits of rehabilitation provided by speech therapists defining subjectively which skills should be the focus of intervention. A promising approach to OPISCoS validation could consist of studies using the OPISCoS form as a basis for measuring progress in response to intervention when combined with criterion outcomes such as the ASQ. Also, we could improve our knowledge of what we know about the benefits of an intervention targeted to communication in children with NDD. Future OPISCoS research is needed to enhance programs for high-risk, atypically developing children.

### Limitations

Some limitations in the current work should be recognized. First, the present paper should be considered a clinical report, and, as a consequence, it has limited generalizability. This kind of study design appears to be adequate to corroborate evidence from clinical experience. In the future, randomized studies are needed to assess the effectiveness of the OPISCoS form in clinical settings with infants diagnosed with NDD and their parents. Second, the implementation of the OPISCoS form in clinical practice is still at the very beginning and no data on the OPISCoS psychometric properties are available yet, limiting the contribution of the present study for scientific research. Although future studies are needed, we found it useful to share our preliminary findings with the clinical and scientific community. Third, infants with NDD may present vast individual differences in their socio-communicative behavior and also caregivers’ behavior could vary considerably across families. Of course, infant's characteristics and parenting patterns could differ depending on the child's clinical conditions and degree of disability and developmental impairments. The OPISCoS form might not be used with infants presenting with very severe NDD, also limiting an observational assessment or an *ad hoc* rehabilitation intervention. Last but not least, this observational procedure in clinical settings should be performed only by practitioners with the relevant expertise and, more generally, experienced in socio-communicative behavioral assessment and parental support for families with NDD infants.

We hope that the use of the OPISCoS form, along with the routine assessment of NDD infants, could help practitioners optimize parenting support interventions and inform clinical services on the possible strategies to improve development in these infants. Our key message is that the difference in interventions for children with NDD is not made only by what we do for children but, above all, by what support we provide to their parents.

## Data Availability

The original contributions presented in the study are included in the article/Supplementary Material, further inquiries can be directed to the corresponding author.
